# Supplementary material for: Determination of Biologically Active Compounds and Antioxidant Capacity In Vitro in Fruit of Small Cranberries (Vaccinium oxycoccos L.) Growing in Natural Habitats in Lithuania
Source: Antioxidants (Basel). 2024 Aug 28;13(9):1045. doi: 10.3390/antiox13091045 (PMC11428458; doi:10.3390/antiox13091045)
Supplement: Supplementary file 1 [file antioxidants-13-01045-s001.zip › antioxidants-3148204-supplementary.pdf]

Supplementary material:

Table S1. Parameters of methods for determining antioxidant capacity and total proanthocyanidin content.

Method	Equivalent compound	Linearity range	Calibration equation	R^2
ABTS•+ Radical Cation Decolorization Assay	Trolox	375-3000 µmol/g	$y = 0.0002\,x – 0.0464$	0.9984
FRAP Assay	Trolox	375-3000 µmol/g	$y = 0.0003\,x – 0.0046$	0.9929
DMCA	Epicatechin	0.0625–1 mg/ml	$y = 0.7021x + 0.0138$	0.9994

Table S2. Parameters of the identified compounds.

Compound	Linearity range (µg/mL)	Calibration equation	R^2	LOD	LOQ
Delphinidin-3-galactoside	3.91–125.00	$y = 4900\,x – 3480$	0.999	1.15	3.19
Cyanidin-3-galactoside	0.78–125.00	$y = 4940\,x – 2370$	0.999	0.16	0.53
Cyanidin-3-glucoside	3.13–100.00	$y = 4230\,x + 1610$	0.999	0.10	3.07
Cyanidin-3-arabinoside	3.13–100.00	$y = 4800\,x + 2220$	0.999	0.93	3.11
Peonidin-3-galactoside	3.125–100	$y = 5320\,x + 8770$	0.999	0.35	2.53
Peonidin-3-glucoside	0.98–125.00	$y = 3970\,x – 1430$	0.999	0.30	0.99
Malvidin-3-galactoside	3.13–100.00	$y = 6890\,x + 3110$	0.999	1.00	3.09
Peonidin-3-arabinoside	3.125–100	$y = 5940\,x + 5320$	0.999	0.42	1.36
Cyanidin	0.78–100.00	$y = 10400\,x – 1930$	0.999	0.07	0.24
Malvidin-3-arabinoside	0.78–125.00	$y = 5950\,x + 1590$	0.999	0.35	1.10
Peonidin	1.56–100.00	$y = 7010\,x +1020$	0.999	0.33	1.09
Malvidin	3.13–100.00	$y=1150\,x + 171$	0.999	0.59	1.96
Myricetin-3-galactoside	0.78–100	$y = 3450\,x – 396$	0.999	0.18	0.54
Quercetin-3-galactoside	3.13–200	$y = 4880\,x + 1180$	0.999	1.01	3.06
Quercetin-3-glucoside	3.13–50	$y = 4160\,x – 61{,}7$	0.999	0.92	2.78
Quercetin-3-α-L-arabinopyranoside	3.13–50	$y = 5250\,x + 861$	0.999	0.70	2.12
Quercetin-3-α-L-arabinofuranoside	3.13–50	$y = 4170\,x – 199$	0.999	0.99	3.03
Quercetin-3-rhamnoside	3.13–50	$y = 3690\,x + 797$	0.999	0.76	2.29
Myricetin	1.56–50	$y = 5360\,x – 1240$	0.999	0.45	1.36
Quercetin	3.13–50	$y = 7450\,x – 1070$	0.999	0.76	2.29
Maslinic acid	3.125–200	$y = 2790\,x + 3990$	0.999	0.89	2.95
Corosolic acid	3.125–200	$y = 3280\,x + 750$	0.999	0.66	2.21
Oleanolic acid	2.344–600	$y = 3240\,x + 12900$	0.999	0.55	1.85
Ursolic acid	3.906–2000	$y = 2930\,x + 39000$	0.999	0.54	1.81
β-Amyrin	6.250–200	$y = 3170\,x + 6470$	0.999	1.11	3.69
α-Amyrin	6.250–200	$y = 3090\,x – 1030$	0.999	1.37	4.58
β-Sitosterol	6.250-200	y = 2100x + 4830	0.9995	1.86	6.18
Squalene	1.563-200	y = 30900x + 50400	0.9999	0.27	0.90
